# Interactive visualization tool to understand and monitor health disparities in diabetes care and outcomes

**DOI:** 10.1017/cts.2024.542

**Published:** 2024-05-17

**Authors:** Jashalynn C. German, Andrew Stirling, Patti Gorgone, Amanda R. Brucker, Angel Huang, Shwetha Dash, David J. Halpern, Nrupen A. Bhavsar, Eugenia R. McPeek Hinz, Richard P. Shannon, Susan E. Spratt, Benjamin A. Goldstein

**Affiliations:** 1 Department of Medicine, Division of Endocrinology, Metabolism, and Nutrition, Duke University School of Medicine, Durham, NC, USA; 2 Duke Health Technology Solutions, Duke University Health System, Durham, NC, USA; 3 Department of Biostatistics and Bioinformatics, Duke University School of Medicine, Durham, NC, USA; 4 Department of Medicine, Division of General Internal Medicine, Duke University School of Medicine, Durham, NC, USA; 5 Duke Primary Care, Duke University Medical Center, Durham, NC, USA; 6 Duke University Health System, Durham, NC, USA; 7 Department of Medicine, Division of Cardiology, Duke University School of Medicine, Durham, NC, USA; 8 Duke Population Health Management Office, Duke Health System, Durham, NC, USA

**Keywords:** Health disparities, real-world data, social determinants of health, type 2 diabetes, visualization

## Abstract

**Objective:**

Type 2 diabetes (T2DM) poses a significant public health challenge, with pronounced disparities in control and outcomes. Social determinants of health (SDoH) significantly contribute to these disparities, affecting healthcare access, neighborhood environments, and social context. We discuss the design, development, and use of an innovative web-based application integrating real-world data (electronic health record and geospatial files), to enhance comprehension of the impact of SDoH on T2 DM health disparities.

**Methods:**

We identified a patient cohort with diabetes from the institutional Diabetes Registry (*N* = 67,699) within the Duke University Health System. Patient-level information (demographics, comorbidities, service utilization, laboratory results, and medications) was extracted to Tableau. Neighborhood-level socioeconomic status was assessed via the Area Deprivation Index (ADI), and geospatial files incorporated additional data related to points of interest (i.e., parks/green space). Interactive Tableau dashboards were developed to understand risk and contextual factors affecting diabetes management at the individual, group, neighborhood, and population levels.

**Results:**

The Tableau-powered digital health tool offers dynamic visualizations, identifying T2DM-related disparities. The dashboard allows for the exploration of contextual factors affecting diabetes management (e.g., food insecurity, built environment) and possesses capabilities to generate targeted patient lists for personalized diabetes care planning.

**Conclusion:**

As part of a broader health equity initiative, this application meets the needs of a diverse range of users. The interactive dashboard, incorporating clinical, sociodemographic, and environmental factors, enhances understanding at various levels and facilitates targeted interventions to address disparities in diabetes care and outcomes. Ultimately, this transformative approach aims to manage SDoH and improve patient care.

## Introduction

Type 2 diabetes mellitus (T2DM) poses a significant public health challenge, effecting over 30 million individuals in the US [1]. Poorly controlled diabetes increases the risk of severe complications, including kidney failure, stroke, visual impairment and blindness, and premature death. Disparities in T2DM management and outcomes disproportionally effect racial and ethnic minority groups, communities with lower socioeconomic status, and inhabitants of rural areas [2–5]. The causes of T2DM-related disparities are multifactorial but have previously been associated with social determinants of health (SDoH) – the conditions in the environments where people are born, live, learn, work, play, worship, and age [6–8]. SDoH can be grouped into five major domains: economic stability, education access and quality, healthcare access and quality, neighborhood and built environments, and social and community context [6].

The “built environment,” which includes the physical characteristics of communities, plays a crucial role in T2DM risk and overall health [7]. Key factors such as access to food, walkability of neighborhoods, and proximity to green spaces/parks significantly influence T2DM incidence and outcomes. The consumption of nutritious food and engagement in physical activity are the fundamental behavioral measures for preventing and managing T2 DM [6,8,9]. Food insecurity, characterized by a lack of consistent access to enough food for a healthy life, is associated with poor dietary quality, cardiometabolic disease onset, and poor control [10,11]. Living in close proximity to fast food restaurants and convenience stores, coupled with a lack of access to grocery stores, contributes to a higher T2DM prevalence, while neighborhoods with green spaces have been linked to lower T2DM risk [8,12–17].

Individuals often simultaneously experience health-related social needs from multiple domains. Many individuals who lack resources in their built environments also face barriers in economic stability and accessing quality healthcare. Populations facing barriers to healthcare access and quality are more likely to be uninsured, have public insurance, receive substandard care, and encounter a multitude of obstacles in accessing care [18]. Studies have shown lack of healthcare facilities in one’s neighborhood can adversely impact access to care, particularly for those relying on public transportation or residing in rural areas [19,20]. Even when individuals facing inequities in access manage to secure health care, studies demonstrate disparities in the quality of care they receive. Notably, prior research has highlighted racial, ethnic, and insurance-based disparities in the use of diabetes medications such as sodium-glucose cotransporter-2 inhibitors (SGLT2i) and glucagon-like peptide-1 receptor agonists (GLP-1RA), drug classes that have been shown to reduce the progression of cardiorenal disease [21]. Additionally, there are racial, ethnic, and insurance-based disparities in the prescribing of continuous glucose monitors, a diabetes technology which has been shown to improve diabetes management [22,23].

The utilization of real-world data, including electronic health records (EHRs) paired with geographical information software, provides an opportunity to understand and monitor not just individual patients, but populations as well [24]. EHRs provide discrete data related to demographics, medications, disease diagnoses, and laboratory results, while geographical information software provides neighborhood spatial files and geocoded locations of points of interest (e.g., grocery stores, medical facilities). However, additional tools are needed to translate these data into easily consumable and actionable information for interested/involved parties [25]. Tableau, a software tool used for data analysis through the use of interactive visualization, can be a key tool to communicate this information [26,27].

In this paper, we report the design, development, and use of an interactive, web-based application that integrates SDoH, patient-reported social risk data, and EHR data. This innovative tool goes beyond conventional methods by offering dynamic visualizations aimed at enhancing comprehension and monitoring capabilities for patients with diabetes within a health system – operating seamlessly across population, neighborhood, group, and individual levels. Our overarching objective is to enhance comprehension and, ultimately, transform the management of SDoH and individual social risk factors to improve patient care. To achieve this, we highlight various clinical, sociodemographic, and neighborhood-level SDoH within the context of a diverse patient population.

## Materials and methods

### Environment

Duke University Health System (DUHS) is a quaternary care academic healthcare system comprising over 400 outpatient clinics and three hospitals in Durham and surrounding counties of North Carolina (NC). DUHS has used an integrated Epic system (Verona, Wisconsin) since 2012. Functioning as the primary healthcare provider in Durham, NC, DUHS provides care for an estimated 86% of individuals in the county [28]. Durham has a unique demographic landscape – with a population of 326,000, featuring a significant representation of racial and ethnic minorities and encompassing a range of urban, suburban, and rural areas. Within this diverse setting, 13% of individuals fall under the federal poverty level; with an even greater number of individuals within the DUHS catchment area experiencing various other forms of SDoH, further emphasizing the complex healthcare needs within our region [29].

### Health disparities analytics program

In 2021, DUHS launched a quality-control initiative to understand and address health disparity across the health system: Collaborative to Advance Clinical Health Equity (CACHE). This program systematically harnesses the power of data science to identify and eliminate disparities in healthcare access and outcomes, focusing on seven domains: race and ethnicity groups, sex, age, neighborhood (defined by “block groups,” the geographical unit used by the United States Census Bureau), insurance status, comorbidities, and patient-reported outcomes. Each project is completed by a multidisciplinary team comprised of clinicians, informaticists, biostatisticians/data scientists, epidemiologists, and a project manager.

Since the program’s inception, CACHE has prioritized the assessment of six significant health domains for potential disparities: maternal morbidity & mortality, hypertension, gun violence, colorectal cancer, prostate cancer, and diabetes. In addition to rigorous analytics, a key component of each project involves the creation of a Tableau dashboard, which facilitates ongoing monitoring of patient populations and guidance for targeted interventions. This paper illustrates the key themes of the diabetes dashboard and provides an in-depth report on our experience in creating this visualization tool, which integrates SDoH and real-world data.

### Eligible population

To identify our population of interest, we began with all adult individuals in the DUHS Epic-based diabetes registry. The diabetes registry employs a comprehensive case definition, including individuals with either an active problem list diagnosis of diabetes, two health system encounters in the past 730 days associated with a billing diagnosis of diabetes, or the presence of an antihyperglycemic agent on the medication list (excluding metformin or GLP-1 classes). Additionally, to be labeled as “active” in the registry an individual must be alive and have had an encounter in the past three years or be in the accountable care organization registry or have a scheduled appointment within the upcoming six months. Notably, patients cannot concurrently have a diagnosis of prediabetes on the problem list to be considered active in the diabetes registry.

Upon further examination of the active registry, we noted the institutional diabetes registry definition is intentionally overly broad (sensitive) and includes patients with diabetes who received inpatient care within DUHS but receive routine outpatient diabetes care elsewhere. Recognizing the need for a more refined definition for our population health surveillance work that has the primary goal of describing the population of patients receiving routine T2DM care within our institution, as an initial step in guiding institutional policies to improve management and outcomes for patients with social risk factors. To ensure the specificity of our surveillance population, we limited our focus to patients with at least one outpatient hemoglobin A1c (HbA1c) measurement in the prior two years as a method to remove patients who may have presented to DUHS for other specialty care but receive T2DM management within a different medical system. In this report, we describe the patient population characteristics based on their eligibility as of June 30, 2022.

### Patient data

For all patients, we extracted clinical information focusing on sociodemographic aspects (age, sex, race, ethnicity, county of residence, insurance payer), comorbidities, service utilization (including clinic type, MyChart [Epic Systems Corporation, Verona, Wisconsin] status), as well as pertinent laboratory results and medications. Detailed variable definitions are presented in Supplemental Table 1. Of particular interest were SDoH factors. Individual-level social risk factors were assessed by leveraging EHR-based SDoH screening results (supplemental Table 2), while neighborhood-level socioeconomic factors were summarized by using state rankings of the Area Deprivation Index (ADI). The ADI, created by a research team at the University of Wisconsin, is a widely used, publicly available, composite measure incorporating: income, employment, education, and poverty levels to establish state-level ranks of census block groups, assigning values from 1 to 10 for each area (with 10 being the highest level of disadvantage) [30]. Since, 2018, DUHS has captured health-related social needs – such as food insecurity, housing insecurity, and transportation challenges – within the dedicated social history section of the EHR. To facilitate comprehensive analysis, patient data were organized into a relational research data mart.

### Descriptive analysis

We systematically assess the patient population across the seven domains of interest: race/ethnicity, sex, age, neighborhood, insurance status, comorbidities, and patient-reported outcomes. To discern potential health disparities, we stratified and compared the patient population based on HbA1c levels: out of control (mean HbA1c ≥ 8%), in control (all HbA1c < 8%), and not measured. We summarized demographic and clinical characteristics, utilizing the standardized mean difference to assess differences across subgroups.

### Visualization design

To enhance comprehensive understanding of the surveillance population among interested/involved parties, we have developed an interactive Tableau dashboard. Our design is tailored to accommodate a diverse range of end-users including researchers, clinicians, care managers, operations staff, and community partners. The backend database view, updated monthly, incorporates data from multiple domains: clinical and individual socioeconomic data within Epic, socioeconomic information from the ADI, and spatial files depicting neighborhood structures and geocoded locations of points of interest. All spatial polygons on the maps in the visualizations are block groups. By integrating several forms of diverse, real-world data into a unified source, we have provided a robust foundation for the Tableau deliverable. The visualizations, as detailed below, aim to foster a comprehensive understanding of our patient cohort at different levels – spanning from the population and neighborhood to groups and, ultimately, the individual.

This work was approved as exempt by the DUHS Institutional Review Board (Pro00111586) and follows the Declaration of Helsinki.

## Results

### Cohort description

**Figure 1.**
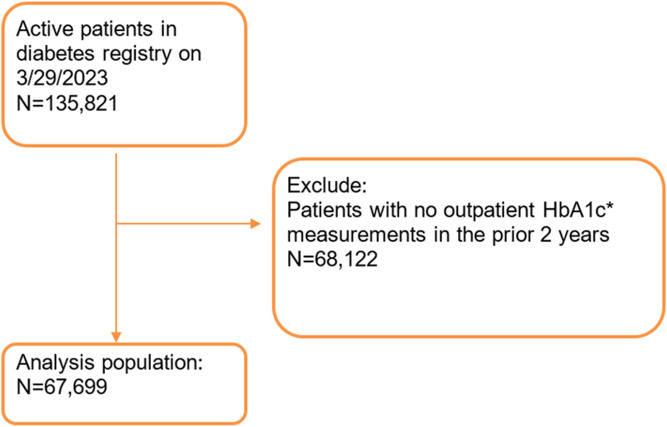
Diagram of analysis population derivation. *HbA1c= hemoglobin A1c.

Based on eligibility criteria, we identified 135,821 active adult patients in the diabetes registry. After excluding 68,122 patients without outpatient HbA1c measurements during our period of interest, we established a cohort of 67,699 individuals for ambulatory diabetes surveillance (Fig. 1). The characteristics of these surveillance population are detailed in Table 1, stratified by HbA1c in the year prior to meeting the eligibility criteria. Of patients with HbA1c values in the previous year, 34% had poorly controlled diabetes (HbA1c≥8%). Based on patient-reported SDoH data, 37% of patients indicated some level of financial strain, 5% reported lack of transportation had kept them from medical appointments or from getting medications, 11% reported worrying that their food would run out before having money to buy more and 9% reported running out of food before having money to buy more. The full list of social risk factor screening questions is given in supplemental Table 2. Additionally, patient characteristics were stratified by ADI level, as outlined in supplemental Table 3. Patients who do not reside in NC but receive diabetes care at Duke and patients who have a PO box listed as their primary address are categorized as “No ADI.”

**Table 1.** Characteristics of the surveillance population stratified by HbA1c

Characteristics	All patients: *N* = 67,699	HbA1c > = 8% in previous year: *N* = 19,204	HbA1c < 8% in previous year: *N* = 36,863	No HbA1c in previous year: *N* = 11,632	Standardized Mean Difference
Age, median (Q1, Q3)	64 (53, 73)	60 (50, 69)	67 (57, 75)	61 (50, 71)	0.43
Sex, female	34,585 (51%)	9614 (50%)	19,263 (52%)	5708 (49%)	0.06
Race/ethnicity					0.26
Non-Hispanic White	33,783 (50%)	8325 (43%)	19,746 (54%)	5712 (49%)	
Non-Hispanic Black	24,554 (35%)	7689 (40%)	12,780 (35%)	4085 (35%)	
Hispanic	4800 (7%)	2021 (11%)	1904 (5%)	875 (8%)	
Non-Hispanic Asian	2571 (4%)	627 (3%)	1518 (4%)	426 (4%)	
Other	1991 (3%)	542 (3%)	915 (2%)	534 (5%)	
Primary payer					0.39
Commercial	21,661 (32%)	6576 (34%)	11,261 (31%)	3824 (33%)	
Dual eligible	5497 (8%)	1729 (9%)	2835 (8%)	933 (8%)	
Medicaid	4230 (6%)	1729 (9%)	1538 (4%)	963 (8%)	
Medicare	13,631 (20%)	2778 (14%)	8718 (24%)	2135 (18%)	
Medicare advantage	15,138 (22%)	3576 (19%)	9514 (26%)	2048 (18%)	
Other	1109 (2%)	366 (2%)	453 (1%)	290 (2%)	
Self-pay	6433 (10%)	2450 (13%)	2544 (7%)	1439 (12%)	
Comorbidities					
Type 1 DM	6709 (10%)	2915 (15%)	2884 (8%)	910 (8%)	0.23
Hypertension	55,209 (82%)	15,532 (81%)	31,334 (85%)	8343 (72%)	0.33
ASCVD	18,033 (27%)	4893 (25%)	10,342 (28%)	2798 (24%)	0.09
Ischemic heart disease	10,744 (16%)	3105 (16%)	6018 (16%)	1621 (14%)	0.07
Stroke	4896 (7%)	1405 (7%)	2769 (8%)	722 (6%)	0.05
Diabetic renal disease	19,308 (29%)	5909 (31%)	10,859 (29%)	2540 (22%)	0.2
ESRD	2830 (4%)	742 (4%)	1526 (4%)	562 (5%)	0.05
Diabetic retinopathy	7157 (11%)	3132 (16%)	3220 (9%)	805 (7%)	0.3
Gastroparesis	1590 (2%)	747 (4%)	625 (2%)	218 (2%)	0.13
Peripheral vascular Disease	7360 (11%)	2196 (11%)	4139 (11%)	1025 (9%)	0.09
Neuropathy	30,222 (45%)	9269 (48%)	17,200 (47%)	3753 (32%)	0.33
Diabetic ketoacidosis	1730 (3%)	1013 (5%)	466 (1%)	251 (2%)	0.23
Alcohol abuse	2160 (3%)	703 (4%)	1104 (3%)	353 (3%)	0.04
Pancreatitis	1781 (3%)	667 (3%)	834 (2%)	280 (2%)	0.07
Congestive heart failure	9186 (14%)	2548 (13%)	5018 (14%)	1620 (14%)	0.02
Osteoporosis	4459 (7%)	933 (5%)	3056 (8%)	470 (4%)	0.18
Recurrent UTI	1161 (2%)	338 (2%)	668 (2%)	155 (1%)	0.04
Diabetic foot ulcer	1778 (3%)	812 (4%)	739 (2%)	227 (2%)	0.13
Amputation	687 (1%)	283 (1%)	304 (1%)	100 (1%)	0.06
Area Deprivation Index, N (%) non-missing	*N* = 49,269 (73%)	13,975 (73%)	*N* = 28,225 (77%)	*N* = 7069 (61%)	
Median (Q1, Q3)	4 (2, 7)	4 (2, 7)	4 (2, 6)	4 (2, 7)	0.15
Financial strain, N (%) non-missing	*N* = 11,637 (17%)	*N* = 3416 (18%)	*N* = 6216 (17%)	*N* = 2005 (17%)	0.27
Hard	2120 (18%)	831 (24%)	908 (15%)	381 (19%)	
Not very hard	2154 (19%)	610 (18%)	1156 (19%)	388 (19%)	
Not hard at all	6827 (59%)	1783 (52%)	3893 (63%)	1151 (57%)	
Decline	536 (5%)	192 (6%)	259 (4%)	85 (4%)	
Food insecurity worry, N (%) non-missing	*N* = 12,232 (18%)	*N* = 3610 (19%)	*N* = 6495 (18%)	*N* = 2127 (18%)	0.22
True	1363 (11%)	556 (15%)	549 (8%)	258 (12%)	
Never true	10,282 (84%)	2862 (79%)	5645 (87%)	1775 (83%)	
Decline	587 (5%)	192 (5%)	301 (5%)	94 (4%)	
Food insecurity ability to pay, N (%) non-missing	*N* = 12,151 (18%)	*N* = 3582 (19%)	*N* = 6461 (18%)	*N* = 2108 (18%)	0.21
True	1128 (9%)	465 (13%)	450 (7%)	213 (10%)	
Never true	10,421 (86%)	2917 (81%)	5706 (88%)	1798 (85%)	
Decline	602 (5%)	200 (6%)	305 (5%)	97 (5%)	
Transportation medical appt, N (%) non-missing	*N* = 11,922 (18%)	*N* = 3503 (18%)	*N* = 6332 (17%)	*N* = 2087 (18%)	0.16
Yes	574 (5%)	251 (7%)	231 (4%)	92 (4%)	
No	11,032 (93%)	3144 (90%)	5947 (94%)	1941 (93%)	
Decline	316 (3%)	108 (3%)	154 (2%)	54 (3%)	
Transportation daily living, N (%) non-missing	*N* = 11,857 (18%)	*N* = 3469 (18%)	*N* = 6315 (17%)	*N* = 2073 (18%)	0.14
Yes	471 (4%)	199 (6%)	194 (3%)	78 (4%)	
No	11,043 (93%)	3153 (91%)	5954 (94%)	1936 (93%)	
Decline	343 (3%)	117 (3%)	167 (3%)	59 (3%)	

HbA1c= hemoglobin A1c; DM = diabetes mellitus; ASCVD = atherosclerotic cardiovascular disease; ESRD = end-stage renal disease; UTI = urinary tract infection.

### Visualization themes

The interactive Tableau dashboard offers enhanced insights into a diverse cohort of patients with T2DM at the population, neighborhood, group, and individual levels.

#### Population visualization

**Figure 2.**
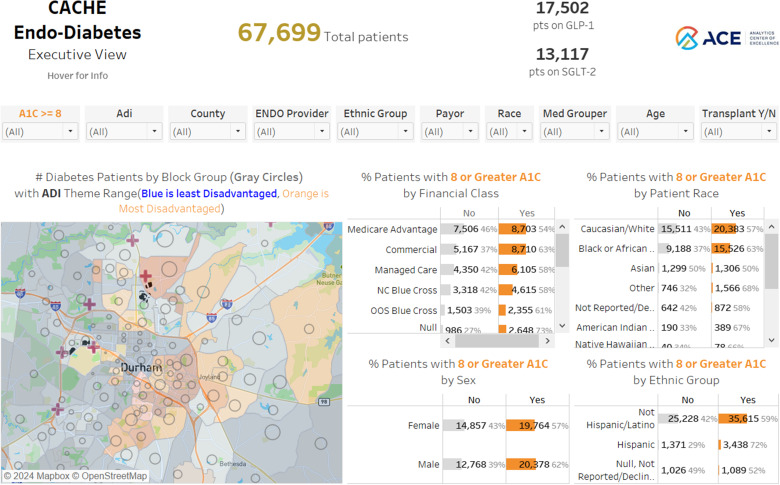
Population-level visualization.

The dashboard’s first objective is to provide a broad overview of the patient population. Figure 2 illustrates the surveillance population living in Durham, NC, and the surrounding counties, offering a geospatial representation with key demographic breakdowns, including sex, race, ethnicity, and insurance status. Environmental socioeconomic status is also depicted through ADI quartiles. Filtering metrics are embedded for clinical domains such as the most recent HbA1c value, county of residency, and provider specialty. During the period from January 1, 2018, to March 29, 2023, 60% (N = 38,194) of the surveillance population had poorly controlled diabetes, defined as a HbA1c ≥ 8%.

#### Neighborhood visualization

**Figure 3.**
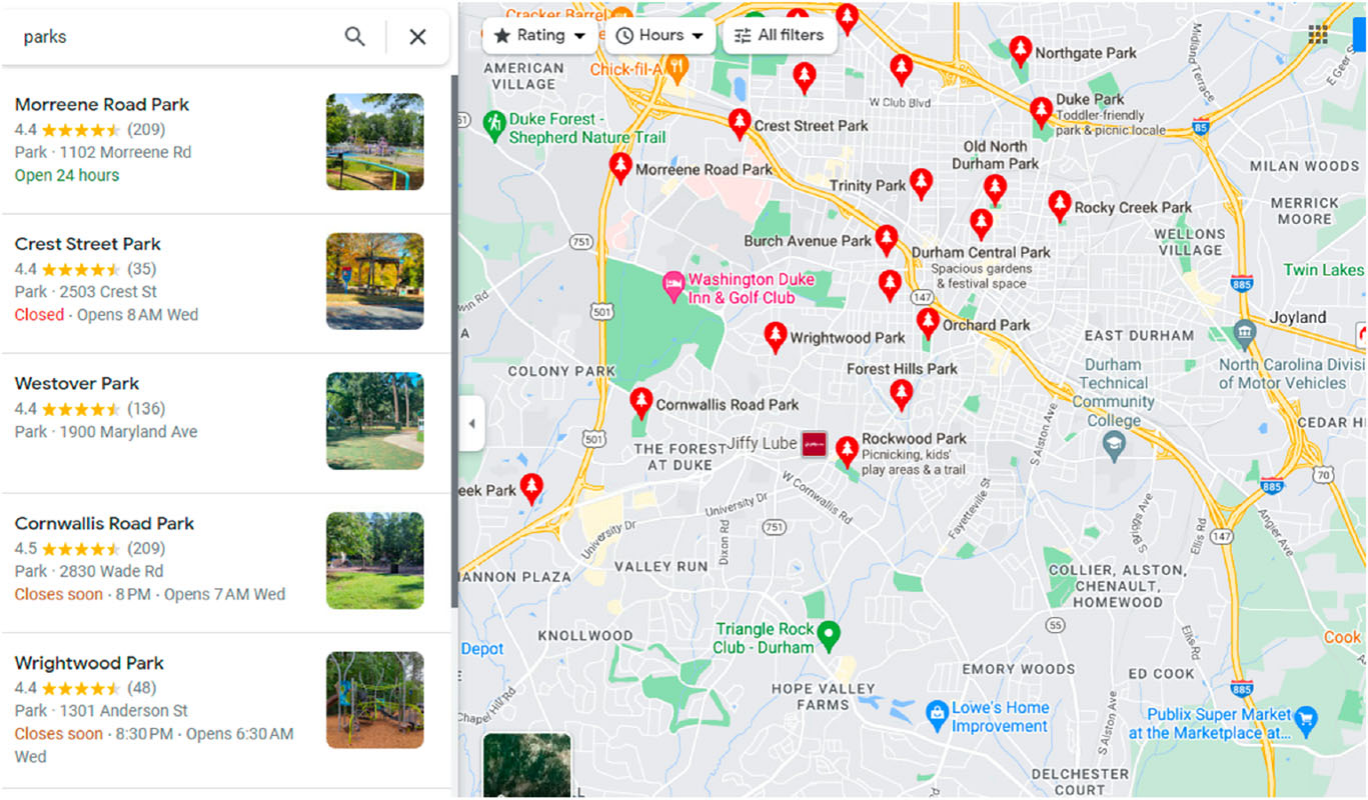
Neighborhood-level visualization.

The neighborhood-level visualization allows users to assess resources and barriers to diabetes care within patients’ neighborhoods (defined by “block groups”). Figure 3 illustrates this capability, wherein we examine the locations of parks within Durham (the largest catchment area for DUHS). Notably, the visualization exposes stark disparities in park distribution across the area, with fewer parks located in areas characterized by higher ADI quartiles. In addition to parks, this level of visualization also allows users to explore various points of interest, ranging from grocery stores to medical clinics, which is of upmost importance as regular physical activity, healthy eating, and routine medical care are crucial to T2DM management.

#### Group-level visualization

**Figure 4.**
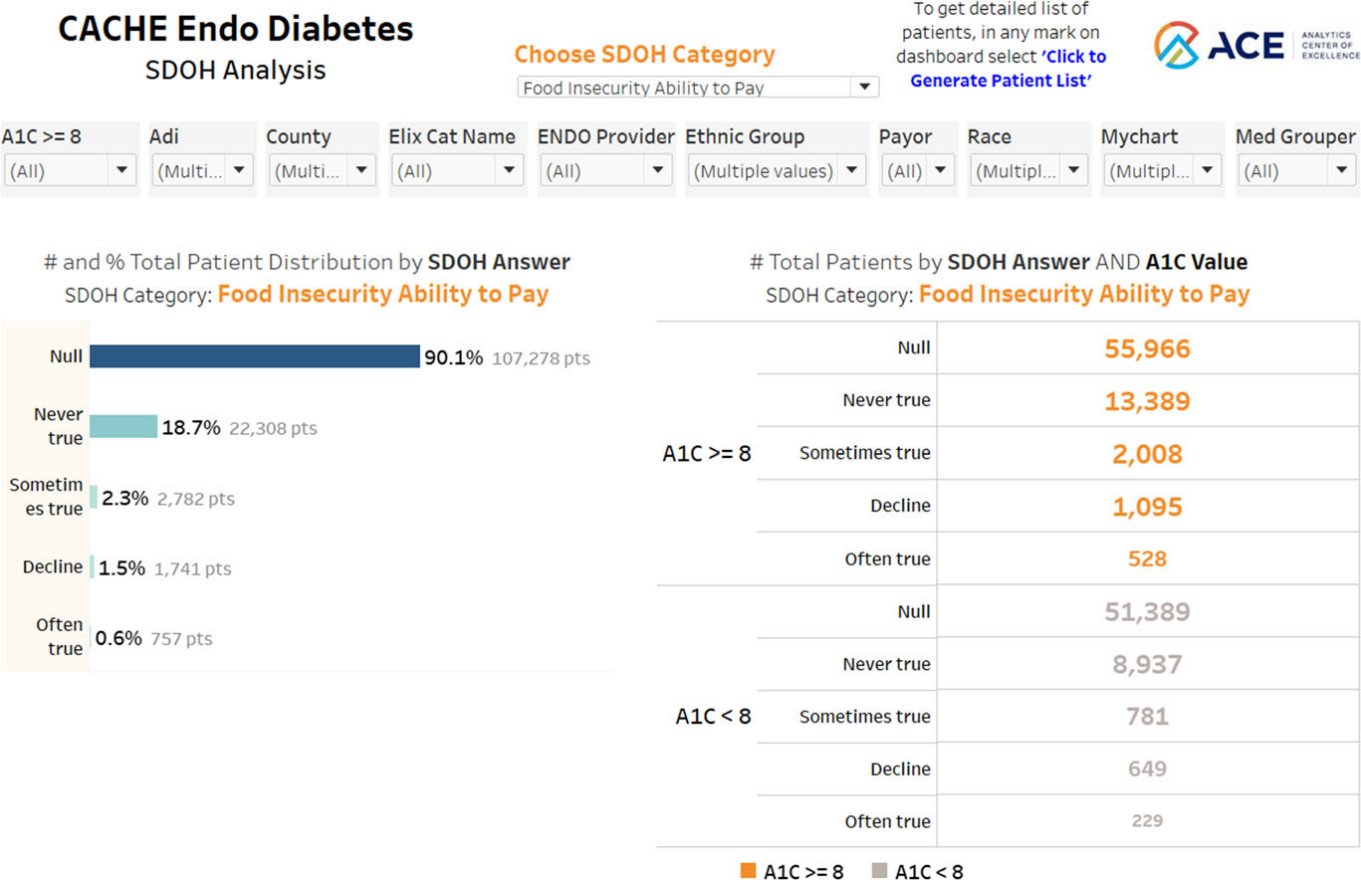
Group-level visualization.

To understand the prevalence of specific patient-reported social risk factors in patients with diabetes, Figure 4 presents the overall rates of patients reporting food insecurity as “inability to pay for food” and stratifies patients based on HbA1c. Groups with a HbA1c ≥ 8% had higher rates of food insecurity compared to patient groups with HbA1c < 8%. The same trend was seen when analyzing groups that reported food insecurity defined as “worry of running out of food” (not shown in Fig. 4). Additional social risk factors are available via a drop-down menu including financial strain and unmet transportation needs. The group-level visualization also provides key clinical and demographic breakdowns, including age, race, ethnicity, medical provider, insurance payor, and ADI.

**Figure 5.**
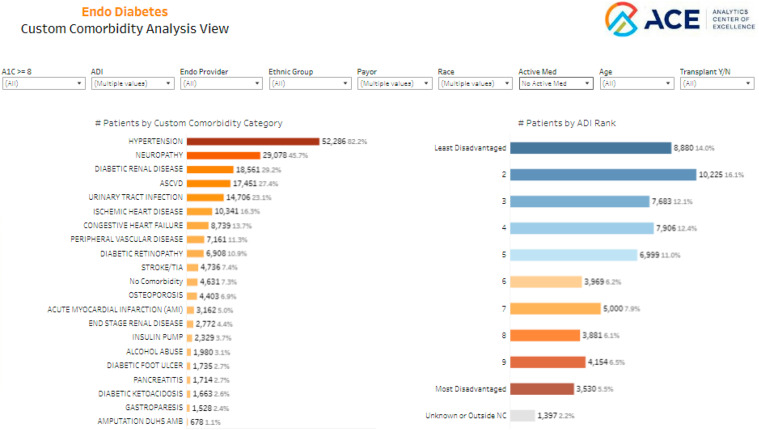
Group-level visualization.

Figure 5 (Custom Comorbidity Analysis) illustrates patients stratified based on comorbidities to assess the increased risk of complications in patients with diabetes. This view not only highlights the prevalence of common clinical comorbidities, diabetes-related complications, and contraindications that may guide the selection of antihyperglycemic medication regimens but also provides key clinical and demographic breakdowns, including A1c control, age, sex, race, ethnicity, insurance payor, and active prescription for SGLT2i or GLP-1RA. In Figure 5, we have filtered the surveillance population for patients not prescribed SGLT2i or GLP-1RA, which revealed significant missed opportunities for prescribing in patients with compelling indications, with 29% having diabetic kidney disease, 27% having atherosclerotic cardiovascular disease (ASCVD), and 14% with congestive heart failure.

#### Individual-level visualization

**Figure 6.**
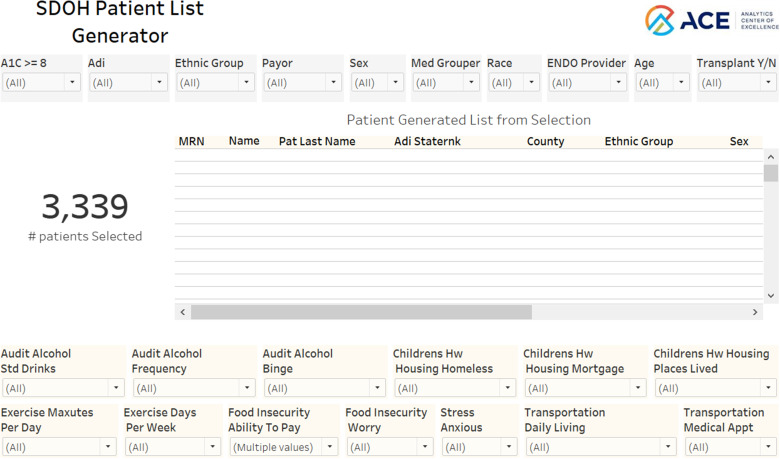
Individual-level visualization.

In Figure 6, the individual-level view allows users to identify specific patient subsets based on demographic, social, and clinical factors. This user-friendly interface allows for the selection of various patient-reported social risk factors (supplemental Table 2) and filtering options, including individual (sex, race, ethnicity, insurance payor) and neighborhood (ADI quartiles) factors. Furthermore, users can also identify patient’s medical providers. Utilizing this view, we identify 3,339 individuals who reported an inability to afford food.

## Discussion

In this paper, we detail the design, development, and utilization of an interactive web-based application designed to seamlessly integrate SDoH, social risk factors, and EHR data into a dynamic visualization tool. The Tableau dashboard leverages granular clinical and demographic data of individuals with diabetes, combined with neighborhood-level geographic, and individual social variables. This comprehensive approach not only enriches monitoring capabilities but also holds the promise of fundamentally reshaping the management of SDoH and elevating the standard of care for patients with diabetes within a large quaternary health system. Operating at various levels – population, neighborhood, group, and individual – this digital health technology offers user-friendly visualizations tailored to a diverse group of users, fostering a more accessible understanding of complex healthcare data. A key emphasis of this dashboard is its role in improving understanding of how SDoH and individual social risk factors impact a medical institution’s patient population and allows investigation into their effects among specific demographic subgroups (e.g., age, race, ethnicity, and insurance payer).

The population level of the dashboard offers a broad overview of differences in diabetes prevalence and control within the surveillance population. Through geospatial representation and environmental socioeconomic status depicted through ADI quartiles, the dashboard highlights several health disparities including a higher prevalence of T2DM within areas with higher ADI and higher rates of poorly controlled T2DM among racial and ethnic minority groups, male sex, and uninsured patients. This high-level visualization holds considerable value for diverse user groups. Health system leadership can leverage it for strategic planning, gaining insights into broad disparities in diabetes prevalence and control. Researchers exploring population-level data for studies on diabetes trends and disparities will find this tool beneficial. Additionally, public health officials and researchers investigating the impact of environmental factors on diabetes within neighborhoods can extract valuable insights from this visualization.

At the neighborhood level, the dashboard highlights contextual factors influencing diabetes management and outcomes within patients’ living environments. Using this view, we saw that neighborhoods with higher ADI had fewer resources crucial to T2DM management including parks/green spaces and grocery stores compared to neighborhoods with lower ADI quartiles. Potential users for this level of visualization include community health organizations interested in contextual factors influencing diabetes management in specific neighborhoods. Local government entities who are concerned with community-wide health outcomes can use this dashboard for potential areas for intervention. Health educators can gain insights into community-specific health challenges to tailor educational programs.

The group-level visualization allows for improved understanding of patient groups based on important clinical grouping factors such as domains of patient-reported social risk factors or comorbidities. Our team utilized this visualization tool to identify missed opportunities for the use of antihyperglycemic medications with cardiorenal protection (i.e., SGLT2 and GLP-1) in patients with compelling medical indications. This level of visualization caters to medical providers interested in understanding patient groups based on clinical or SDoH groupings, care managers seeking insights into specific patient groups for targeted care coordination, and researchers investigating correlations between clinical and SDoH factors in specific demographic subgroups.

At the most granular view, individual level, this visualization tool also aids in generating patient lists, sorted by various clinical, demographic, and social risk factor characteristics, which allow users to gain improved understanding of individuals. Potential users for this level of visualization include clinicians or researchers interested in generating patient lists to identify patients for targeted therapies/interventions based on clinical, demographic, and SDoH characteristics. Care managers or clinic leadership can use this individual-level data for personalized care planning and support of clinic populations. Our team has utilized the capabilities of the dashboard to identify and recruit individuals with diabetes and who are at risk for food insecurity to participate in an ongoing randomized control trial, Eat Well. This 12-month, two-arm trial aims to enhance diabetes self-management through educational resources including nutritional guidance. All participants receive information about existing care management resources, while the intervention arm additionally receives a monthly food voucher for purchasing fruits and vegetables. The Eat Well trial has successfully enrolled over 2000 patients. Outcomes of interest include glycemic control, changes in weight, lipid panel, blood pressure, and utilization rates of the provided vouchers.

The utilization of this visualization tool has yielded findings that align with prior research, demonstrating a correlation between SDoH, individual social risk factors, and disease burden, particularly poorly controlled diabetes and its related complications [8,9,12,16,17,31]. Previous studies investigating the influence of SDoH domains and social risk factors on diabetes are constrained by limitations such as the absence of individual-level data or the potential for same-source bias, often relying solely on patient-reported data. Our work is innovative as it incorporates multilevel real-world data, including neighborhood-level variables (ADI and geospatial data), as well as individual-level social risk factor data. The integration of multidimensional variables into the current dashboard presents descriptive associations that suggest potential inequities in diabetes incidence, the delivery of care, and outcomes. While clinical and operationally oriented end-users use this dashboard to gain an understanding of how patient care is being delivered, researchers are also using the dashboard to generate hypotheses to motivate more targeted analyses related to potential health disparities. For example, we have leveraged the source data from the presented dashboard to investigate differences in prescribing of sodium-glucose cotransporter-2 inhibitors (SGLT2i) and glucagon-like peptide-1 receptor agonists (GLP-1RA) medications and whether there is evidence of therapeutic inertia (failure to initiate or intensify therapy when therapeutic goals are not met). Other potential questions, generated from the descriptive findings of this dashboard, include investigating geographic variations in diabetes management practices and outcomes within the healthcare system and identifying disparities in healthcare utilization. In total, such a dashboard allows both clinically oriented users to quickly understand care delivery, while research-oriented users can use it as a springboard to motivate future investigation.

While the presented dashboard features have clear scientific purpose for healthcare system leadership, clinicians, care managers, health educators, and researchers by enhancing the understanding of patient populations and the role of SDoH in health disparities, its utility extends beyond. This dynamic digital health technology holds potential significance for both public entities (e.g., local government) and private sectors (e.g., insurance payor) as it may prompt targeted policy changes aimed at alleviating environmental and social barriers that contribute to health disparities. Examples include improving access to produce by expanding Supplemental Nutrition Assistance Program benefits or advocating for legislative policies that enhance the built environment (equitable distribution of grocery stores and parks across cities).

As a health system, we recognize the importance of understanding and supporting our local community, particularly those facing heightened health risks. The CACHE initiative, the foundation of our current work, represents a concerted effort to understand sources of health disparity and propose viable solutions. Visualization tools, integral to this initiative, empower users to gain a deeper understanding of the underlying factors leading to disparities in care and outcomes. We have previously highlighted CACHE’s utilization of Tableau dashboards to enhance hospital operations and display clinical decision support tools [26,32]. In prior usage, Tableau dashboards have proven advantageous, creating simple, easily interpretable visualizations – a crucial factor given that many of these dashboards are accessed by non-technical users such as care managers, nurses, and operations staff. Recognizing the significance of population health maps and visualizations we acknowledge their pivotal role in enhancing our comprehension of patient health [24,33].

While the presented work is significant and impactful, we do recognize some limitations. This work reflects the experience of a single institution, and the replicability of this approach may be contingent on the presence of supportive health information technology (IT) and data science teams to undertake the time-consuming and labor-intensive nature of building Tableau dashboards. DUHS represents a unique example as the institution has the support of health IT and data science specialist tightly integrated into the needs of hospital and clinical operations, and Tableau has been utilized within the institution since 2016, which provides necessary technical expertise to develop these tools and end-user literacy and comfort to effectively utilize the software platform. Despite our institution’s experience with creating these innovative visualization tools, we recognize the need for ongoing efforts to make these tools more accessible and actionable. EHR integration and privacy hurdles are acknowledged. Currently, the Tableau dashboard is not directly integrated into the EHR, so users interested in contacting a patient or medical provider for intervention would need to do so outside of the dashboard; however, the dashboard is guiding the development of clinical decision support within the EHR. Direct access to the Tableau dashboard by community partners is restricted given the incorporation of protected health information; however, we have been able to generate summary dashboards of community-based health indicators that are available to public partners [34]. While this work highlights the dashboard’s incorporation of several social risk factors (food insecurity, transportation difficulties, and financial strain), we recognize that there are a multitude of social risk factors that may affect the surveillance population that are not currently captured within our health system’s social risk screening tool. Future work will seek to gather additional patient social risk factors. Additionally, while ADI is a useful tool for assessing socioeconomic deprivation at the neighborhood level, we recognized it does not comprehensively capture all dimensions of deprivation, as it does not account for important factors such as cultural, behavioral, aspects that contribute to health disparities. Future work will seek to gather additional neighborhood-level socioeconomic status factors. As we navigate these complexities, the overarching goal of our work is to empower users to better understand the role of SDoH and social risk factors in health disparities, contributing to the broader mission of the CACHE initiative.

## Conclusion

We demonstrate the successful integration of patient-reported SDoH data and real-world data (EHR and geospatial files) within our institution to create an interactive web-based application. This digital health tool utilizes visualization to enhance understanding of how SDoH and social risk factors impact patients with diabetes. These visual aids play a pivotal role in making complex health data accessible to a diverse range of users. The design and utilization of this easy-to-use web-based visualization tool are instrumental in comprehending and addressing the role of SDoH and social risk factors in health disparities and improving health outcomes. Leveraging, Tableau, as our platform of choice, seamlessly embeds clinical, social, and demographic factors into a unified view. This not only enables observation of populations and neighborhoods but also provides insights at group and individual levels, offering actionable items to address patient needs. The culmination of these efforts aims to empower healthcare professionals, researchers, and community partners to proactively address health disparities.

## Supporting information

German et al. supplementary material German et al. supplementary material

## Supplementary material

The supplementary material for this article can be found at https://doi.org/10.1017/cts.2024.542.
