# Supplementary material for: Interactive visualization tool to understand and monitor health disparities in diabetes care and outcomes
Source: J Clin Transl Sci. 2024 May 17;8(1):e102. doi: 10.1017/cts.2024.542 (PMC11362627; doi:10.1017/cts.2024.542)
Supplement: German et al. supplementary material [file S2059866124005429sup001.docx]

**Supplement**

Table 1: Detailed variable definitions

Table 2: Patient Social Risk Factor Screening Questions

Table 3: Characteristics of the surveillance population stratified by ADI

Table 1: Detailed variable definitions

| Grouping | Variable | Definition |
| --- | --- | --- |
| Demographic | Sex | Sex of the patient. For Epic data, this is the legal sex of the patient as defined by a government body.  Male, Female and Unknown |
|  | Age | Patient’s current age |
|  | Race | American Indian or Alaskan Native  Asian  Asian Indian  Black or African American  Caucasian/White  Chinese  Filipino  Guamanian or Chamorro  Japanese  Korean  Native Hawaiian  Native Hawaiian or Other Pacific Islander  Not Reported/Declined  Other  Other Asian  Other Pacific Islander  Samoan  Vietnamese |
|  | BMI | Category: Underweight, Normal, Obese, Overweight |
| Medical information | Patient outcome at discharge | Describe where the patient was discharged after an inpatient or emergency department encounter. |
|  | Admission Type | The description of the admission type (elective surgery, etc.). |
|  | Transfer to ICU | Indicates whether the patient was ever transferred to the ICU during an inpatient stay. |
|  | Encounter Type | Category: Emergency Department, Emergency Department Admit to Inpatient Stay, Inpatient Hospital Stay, Observation Stay |
|  | Length of Stay | Discharge Date minus Admit Date |
|  | Raw payer type value | Payment Information  Category: Private(commercial, managed care, Medicare advantage, NC blue cross, out of state blue cross), Public(Medicaid pending, Medicare, other government, NC Medicaid, NC Medicaid managed care), Self-pay, Other(special programs) |
|  | Vaccine Status | COVID Vaccine Status |
| Comorbidity | Cancer | ICD-10 code: C* |
|  | Cardiovascular | ICD-10 code: I20-I59 |
|  | Hypertension | ICD-10 code: I10-I11 |
|  | Chronic Liver Disease | ICD-10 code: K70-K77 |
|  | Chronic Obstructive Pulmonary Disease | Chronic Obstructive Pulmonary Disease  ICD-10 code: J44 |
|  | ASTHMA | Asthma  ICD-10 code: J45 |
|  | Chronic Renal Disease | ICD-10 code: N18 |
|  | Diabetes | ICD-10 code: E08-E13 |
| Medicine | Bronchodilator | Bronchodilator |
|  | Steroid | Steroid, Corticosteroid |
|  | Anticoagulant Antiplatelet | Anticoagulant |
|  | Diuretic | Diuretic |
|  | Cough Suppressant | Cough Suppressant,  Expectorant with cough suppressant |
|  | Paralytic | Paralytic,  Used during intubation as a paralytic |
|  | Expectorant | Expectorant |
|  | Remdesivir | Remdesivir |
|  | Inhaled.Steroid | Inhaled Steroid |
| Lab test | Lymphocyte.Count..Absolute | The absolute number of Lymphocytes |
|  | Lymphocyte.Count | The number of Lymphocytes |
|  | CRP..C.reactive.Protein..Inflammatory | C-Reactive Protein |
|  | Ferritin | Ferritin level: High, Low, Normal, Not Taken |
|  | D.Dimer | D-Dimer level: High, Normal, Not Taken |
|  | Procalcitonin | Procalcitonin level: High, Missing, Normal |

Table 2: Patient Social Risk Factor Screening Questions

| Alcohol frequency: | - How often do you have a drink containing alcohol? - How many drinks containing alcohol do you have on a typical day when you were drinking? |
| --- | --- |
| Alcohol binge: | - How often do you have six or more drinks on one occasion? |
| Exercise: | - On average, how many days per week do you engage in moderate to strenuous exercise (like a brisk walk)? - On average how many minutes do you engage in exercise at this level? |
| Financial resource strain: | - How hard is it for you to pay for the very basics like food, housing, medical care and heating? |
| Food insecurity ability to pay: | - Within the past 12 months, has the food you bought just didn't last and you didn't have money to get more? |
| Food insecurity worry: | - Within the past 12 months, have you worried that your food would run out before you got money to buy more? |
| Housing homelessness: | - In the past 12 months, was there a time when you do not have a city place to sleep or slept in a shelter (including now)? |
| Housing mortgage: | - In the last 12 months, was there a time when you were not able to pay the mortgage or rent on time? |
| Housing places live: | - In the past 12 months, how many places have you lived |
| Social Support | - How often do you get together with friends or relatives? - How often do you attend church or religious services? - Do you belong to any clubs or organizations such as church groups, unions, fraternal or athletic groups or school groups? - Are you married, widowed, divorce, separated, never married, or living with a partner? - In a typical week, how many times do you talk on the phone with family, friends, or neighbors? |
| Stress/ Anxious: | - Do you feel stress, tense, restless, nervous, or anxious, or unable to sleep at night because your mind is troubled all the time- these days? |
| Transportation daily living: | - In the past 12 months is lack of transportation kept you from meetings, work, or from getting things needed for daily living? |
| Transportation medical appointment: | - In the past 12 months, has lack of transportation kept you from medical appointments or from getting medications? |

Table 3: Characteristics of the surveillance population stratified by ADI

| Characteristics | All patients: N=67699 | ADI 1-2:  N=15770 | ADI 3-4:  N=12591 | ADI 5-6:  N=8588 | ADI 7-8:  N=6843 | ADI 9-10: N=5477 | Standardized Mean Difference |
| --- | --- | --- | --- | --- | --- | --- | --- |
| Age Median (Q1, Q3) | 64 (53, 73) | 65 (55, 74) | 64 (54, 73) | 64 (54, 73) | 64 (54, 73) | 63 (53, 72) | 0.12 |
| Sex Female | 34585 (51%) | 7358 (47%) | 6502 (52%) | 4590 (53%) | 3748 (55%) | 3157 (58%) | 0.22 |
| Primary Payer |  |  |  |  |  |  | 0.61 |
| Commercial | 21661 (32%) | 6357 (40%) | 4577 (36%) | 2783 (32%) | 1900 (28%) | 1338 (24%) |  |
| Dual eligible | 5497 (8%) | 731 (5%) | 851 (7%) | 781 (9%) | 770 (11%) | 867 (16%) |  |
| Medicaid | 4230 (6%) | 608 (4%) | 669 (5%) | 548 (6%) | 553 (8%) | 584 (11%) |  |
| Medicare | 13631 (20%) | 4091 (26%) | 2590 (21%) | 1647 (19%) | 1349 (20%) | 882 (16%) |  |
| Medicare Advantage | 15138 (22%) | 3344 (21%) | 3157 (25%) | 2269 (26%) | 1818 (27%) | 1402 (26%) |  |
| Other | 1109 (2%) | 150 (1%) | 204 (2%) | 165 (2%) | 133 (2%) | 103 (2%) |  |
| Self-Pay | 6433 (10%) | 489 (3%) | 543 (4%) | 395 (5%) | 320 (5%) | 301 (5%) |  |
| Comorbidities |  |  |  |  |  |  |  |
| Type 1 DM | 6709 (10%) | 1784 (11%) | 1186 (9%) | 783 (9%) | 622 (9%) | 525 (10%) | 0.07 |
| Hypertension | 55209 (82%) | 12698 (81%) | 10552 (84%) | 7221 (84%) | 5909 (86%) | 4686 (86%) | 0.16 |
| ASCVD | 18033 (27%) | 4059 (26%) | 3314 (26%) | 2422 (28%) | 1984 (29%) | 1577 (29%) | 0.07 |
| Ischemic heart disease | 10744 (16%) | 2276 (14%) | 2006 (16%) | 1511 (18%) | 1212 (18%) | 1006 (18%) | 0.11 |
| Stroke | 4896 (7%) | 991 (6%) | 909 (7%) | 706 (8%) | 558 (8%) | 486 (9%) | 0.1 |
| Diabetic Renal Disease | 19308 (29%) | 4091 (26%) | 3612 (29%) | 2603 (30%) | 2228 (33%) | 1840 (34%) | 0.17 |
| ESRD | 2830 (4%) | 420 (3%) | 435 (3%) | 381 (4%) | 340 (5%) | 359 (7%) | 0.19 |
| Diabetic Retinopathy | 7157 (11%) | 1691 (11%) | 1354 (11%) | 903 (11%) | 782 (11%) | 700 (13%) | 0.07 |
| Gastroparesis | 1590 (2%) | 273 (2%) | 292 (2%) | 212 (2%) | 186 (3%) | 158 (3%) | 0.08 |
| Peripheral Vascular Disease | 7360 (11%) | 1523 (10%) | 1333 (11%) | 1030 (12%) | 903 (13%) | 710 (13%) | 0.11 |
| Neuropathy | 30222 (45%) | 6740 (43%) | 6000 (48%) | 4270 (50%) | 3406 (50%) | 2643 (48%) | 0.14 |
| Diabetic Ketoacidosis | 1730 (3%) | 331 (2%) | 278 (2%) | 237 (3%) | 197 (3%) | 188 (3%) | 0.08 |
| Alcohol Abuse | 2160 (3%) | 407 (3%) | 346 (3%) | 281 (3%) | 267 (4%) | 227 (4%) | 0.09 |
| Pancreatitis | 1781 (3%) | 369 (2%) | 310 (2%) | 244 (3%) | 219 (3%) | 179 (3%) | 0.06 |
| Congestive Heart Failure | 9186 (14%) | 1720 (11%) | 1593 (13%) | 1215 (14%) | 1050 (15%) | 924 (17%) | 0.17 |
| Osteoporosis | 4459 (7%) | 1215 (8%) | 888 (7%) | 584 (7%) | 471 (7%) | 326 (6%) | 0.07 |
| Recurrent UTI | 1161 (2%) | 302 (2%) | 229 (2%) | 148 (2%) | 112 (2%) | 96 (2%) | 0.02 |
| Diabetic Foot Ulcer | 1778 (3%) | 321 (2%) | 349 (3%) | 259 (3%) | 225 (3%) | 178 (3%) | 0.08 |
| Amputation | 687 (1%) | 85 (1%) | 134 (1%) | 92 (1%) | 95 (1%) | 91 (2%) | 0.11 |
| Financial strain, N (%) non-missing |  |  |  |  |  |  | 0.47 |
| Hard | 2120 (18%) | 439 (12%) | 397 (18%) | 295 (23%) | 242 (25%) | 212 (29%) |  |
| Not very hard | 2154 (19%) | 655 (17%) | 433 (20%) | 232 (18%) | 179 (18%) | 143 (19%) |  |
| Not hard at all | 6827 (59%) | 2494 (66%) | 1238 (57%) | 690 (54%) | 514 (52%) | 348 (47%) |  |
| Decline | 536 (5%) | 166 (4%) | 97 (4%) | 59 (5%) | 48 (5%) | 36 (5%) |  |
| Food insecurity worry, N (%) non-missing |  |  |  |  |  |  | 0.33 |
| True | 1363 (11%) | 263 (7%) | 241 (11%) | 195 (14%) | 168 (16%) | 139 (17%) |  |
| Never true | 10282 (84%) | 3439 (89%) | 1905 (85%) | 1098 (81%) | 829 (79%) | 634 (77%) |  |
| Decline | 587 (5%) | 179 (5%) | 103 (5%) | 67 (5%) | 53 (5%) | 47 (6%) |  |
| Food insecurity ability to pay, N (%) non-missing |  |  |  |  |  |  | 0.34 |
| True | 1128 (9%) | 210 (5%) | 191 (9%) | 157 (12%) | 136 (13%) | 126 (16%) |  |
| Never true | 10421 (86%) | 3461 (90%) | 1942 (87%) | 1124 (83%) | 849 (82%) | 635 (79%) |  |
| Decline | 602 (5%) | 182 (5%) | 107 (5%) | 71 (5%) | 54 (5%) | 47 (6%) |  |
| A1c measurements |  |  |  |  |  |  |  |
| Number of A1c measurements |  |  |  |  |  |  | 0.11 |
| 0 | 11632 (17%) | 2179 (14%) | 1758 (14%) | 1212 (14%) | 1054 (15%) | 866 (16%) |  |
| 1 | 18370 (27%) | 4103 (26%) | 3006 (24%) | 2178 (25%) | 1722 (25%) | 1454 (27%) |  |
| 2 | 20519 (30%) | 5307 (34%) | 4182 (33%) | 2811 (33%) | 2103 (31%) | 1623 (30%) |  |
| 3 | 12366 (18%) | 3039 (19%) | 2669 (21%) | 1716 (20%) | 1399 (20%) | 1056 (19%) |  |
| >=4 | 4812 (7%) | 1142 (7%) | 976 (8%) | 671 (8%) | 565 (8%) | 478 (9%) |  |
| Mean A1c in previous year, Median (Q1, Q3) | 7 (6.4, 8) | 6.9 (6.4, 7.8) | 7 (6.4, 8) | 7 (6.4, 8) | 7.1 (6.4, 8.1) | 7.2 (6.4, 8.3) |  |

DM= diabetes mellitus; ASCVD= atherosclerotic cardiovascular disease; ESRD= end stage renal disease; UTI= urinary tract infection
